# Transsubclavian versus transfemoral TAVR with self-expanding valves

**DOI:** 10.3389/fcvm.2026.1901010

**Published:** 2026-08-19

**Authors:** Funda Baysal, Kira Osipenko, Severin Laengle, Irene Steiner, Amila Kahrovic, Paul Werner, Philipp Bartko, Daniel Zimpfer, Martin Andreas, Iuliana Coti

**Affiliations:** 1 Department of Cardiac and Thoracic Aortic Surgery, Medical University of Vienna, Vienna, Austria; 2 Center for Medical Data Science, Institute of Medical Statistics, Medical University of Vienna, Vienna, Austria; 3 Department of Internal Medicine II, Medical University of Vienna, Vienna, Austria; 4 Department of Cardiac Surgery, Medical University of Graz, Graz, Austria

**Keywords:** alternative access, aortic stenosis, self-expandable prosthesis, transcatheter aortic valve replacement, transsubclavian TAVR

## Abstract

**Introduction:**

Transsubclavian (TS) access has emerged as a rapidly growing alternative to the traditional transfemoral (TF) approach in patients with hostile iliofemoral access undergoing transcatheter aortic valve replacement (TAVR). The aim of this study was to investigate clinical outcomes following TS-TAVR and TF-TAVR in severe aortic valve stenosis treated with self-expanding devices.

**Methods:**

We conducted a single-center retrospective analysis of consecutive patients undergoing TS- or TF-TAVR between August 2017 and May 2025 at our department. Clinical endpoints were reported according to the Valve Academic Research Consortium-3 (VARC 3) criteria.

**Results:**

A total of 817 patients underwent TF-TAVR, while 75 patients underwent TS-TAVR. Patients undergoing TS-TAVR were more frequently men, presented with a higher Euro Score II, and carried a greater burden of atherosclerotic disease. Unadjusted data analysis showed a significant association between TS access and 30-day mortality [HR 2.64, 95% CI: 1.00–6.97, *p* = 0.05], stroke [HR 2.44, 95% CI: 1.19–5.01, *p* = 0.01], myocardial infarction [HR 4.31, 95% CI: 1.16–15.96, *p* = 0.03], major bleeding [HR 1.79, 95% CI: 1.17–2.73, *p* = 0.007], and cardiovascular hospitalization [HR 1.65, 95% CI: 1.02–2.65, *p* = 0.01]. After risk adjustment, the access site remained independently associated with stroke and major bleeding, while 30-day mortality and cardiovascular hospitalization lost statistical significance.

**Conclusion:**

Overall, TS-TAVR appears to be a feasible approach when TF-TAVR is not possible. Adjusted data revealed no statistically significant association with early mortality. However, the access site was significantly associated with stroke and major bleeding, findings likely attributable to the higher baseline risk of the subclavian cohort in our study.

## Introduction

Transcatheter aortic valve replacement (TAVR) was first performed in 2002 as an alternative to surgical aortic valve replacement (SAVR). The establishment of devices with lower insertion profiles resulted in a significant increase in procedures performed via transfemoral access, with major clinical trials showing excellent outcomes for patients across all surgical risk classes compared with SAVR (1). However, considering the advanced age of patients undergoing TAVR, a substantial proportion may present with severe peripheral vessel disease or other relevant limitations—such as severe aortic tortuosity, prior vascular surgery, and small arterial lumen diameter (<5 mm)—thereby precluding iliofemoral access (2).

Alternative access routes have been developed for patients unsuitable for TF-TAVR. Intrathoracic implantation routes such as transapical (TA) and direct transaortic (TAo) approaches are considered invasive techniques, requiring thoracotomy, general anesthesia, and intensive care, shifting interest toward less invasive peripheral routes (3). TS-TAVR is the most attractive alternative to TF-TAVR as the subclavian artery is less affected by severe atherosclerosis and tortuosity, generally has a greater vessel diameter than 5.0 mm, and provides a more direct pathway with a shorter distance from the access site to the aortic annulus, allowing advanced device handling (1).

Although technically more challenging than transfemoral access, previous registry studies have demonstrated the feasibility and safety of TS-TAVR (4, 5). However, given its deep anatomical location requiring extensive surgical dissection, concerns exist regarding access-related major and life-threatening bleeding. Further, some series have also reported an increased risk of cerebrovascular events, possibly due to anatomical proximity to the left carotid artery. Moreover, longer procedural times and increased contrast medium use during TS-TAVR may increase the frequency of postoperative acute kidney injury in multimorbid patients (2, 6).

The choice of alternative access routes varies across institutions and is influenced by both patient-specific factors and institutional preferences. Current guidelines do not provide clear recommendations regarding the optimal alternative access route in TAVR. To the best of our knowledge, no randomized controlled trail has compared major outcomes of subclavian and femoral access. The available evidence is limited to a small number of retrospective studies and meta-analyses, with only partially adjusted data. Moreover, much of this evidence is based on earlier-generation devices. Therefore, adverse events associated with TS-TAVR require further investigation (7).

At our institution, transsubclavian access is the preferred alternative strategy. Herein, we provide our intuitional experience and real-world data on major clinical outcomes following transsubclavian and transfemoral TAVR.

## 2 Methodology

### 2.1 Study design and patients

The present study is a retrospective data analysis of all patients who underwent TS- or TF-TAVR between August 2017 and May 2025 at the Department of Cardiac and Thoracic Aortic Surgery, Medical University of Vienna. This analysis includes only patients treated with self-expanding devices (Abott Navitor und Portico, Medtronic Evolut R, FX, FX+, Allegra, Symetis Acurate). Procedures using the Jena Valve Trilogy transcatheter heart valve (JenaValve Technology)—which is primarily used in patients with isolated severe aortic regurgitation—were excluded. Data collection was conducted retrospectively and prospectively within the framework of the VICTORY-II registry (Ethics Number: 1680/2020), in accordance with the Declaration of Helsinki and Good Clinical Practice, as previously reported.

### 2.2 Access site and procedural aspects

Based on preoperative computed tomography (CT), the interdisciplinary heart team determined the optimal access route for the TAVR procedure. Transfemoral TAVR was the primary access, performed percutaneously—predominantly via the right femoral artery—under conscious sedation. A percutaneously vascular closure system was used for closure of the arterial access site. In cases of unsuitable femoral anatomy (inadequate vessel diameter, severe calcification or occlusion, severe tortuosity or kinking, aneurysm/dissection, and thrombotic appositions at the vessel site or descending aorta), transsubclavian TAVR was the preferred alternative access at our institution.

**Figure 1:**
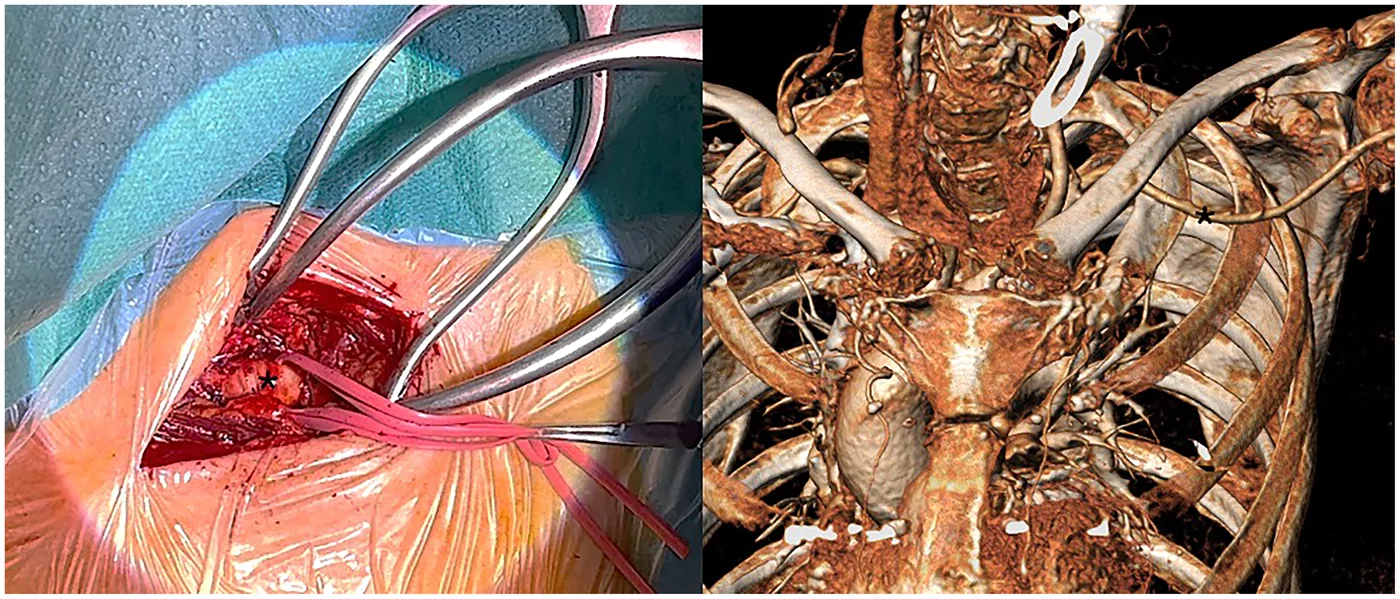
Transsubclavian access site. Left: Surgical field during TS-TAVR; Right: 3D image depicting the anatomical feature of the access site. (*) marks the axillary artery.

Historically, the terms transsubclavian and transaxillary have been distinguished based on the arterial entry site. However, in the contemporary literature, these terms are often used interchangeably, with transsubclavian frequently serving as a misnomer to describe the infraclavicular (i.e., transaxillary) approach rather than true supraclavicular subclavian access. In our study, subclavian access was performed under general anesthesia via an open surgical cut-down through the left infraclavicular area (Figure 1). The left axillary artery was preferred, as it provides a better implantation angle than the right one. For the closure we placed two purse-string sutures before puncture.

Further, the presence of a patent left internal mammary artery (LIMA) was not considered a contraindication in patients undergoing TS-TAVR, provided vessel diameter was sufficient.

### 2.3 Definitions and outcomes

The primary outcome was defined as all-cause mortality at 30 days. Secondary endpoints included clinical events defined according to the VARC 3 criteria, such as major bleeding, major vascular complications, major cardiac structural complications, postoperative myocardial infarction, stroke, new permanent pacemaker implantation, acute kidney injury, and cardiovascular hospitalization. Bleeding was classified into Type 1–4 based on the need for intervention, amount of red blood cell transfusions, hemoglobin drop, end-organ failure, and death. In this analysis, major bleeding events were defined as VARC Type ≥ 2. Perioperative blood transfusion was reported separately.

Vascular complications were routinely assessed via intraoperative angiography and by systematic arterial Doppler examination of the groin after the index procedure. According to the VARC 3 criteria, major vascular complications are defined as those resulting in death, VARC Type ≥ 2 bleeding, limb or visceral ischemia, or irreversible neurologic impairment.

Intraoperative outcomes—such as valve malposition, intraoperative new conduction disturbances, paravalvular leak (PVL) at the end of the procedure, postoperative admission to the intensive care unit (ICU), and the length of hospital stay (LOS)—between the two groups were also studied.

### 2.4 Statistical analysis

Categorical variables were reported as absolute frequencies and percentages, with group comparisons assessed using Fisher's exact tests. Continuous variables with an approximately normal distribution were described using mean ± standard deviation (SD), while non-normally distributed variables were reported as median with interquartile ranges (first quartile Q1; third quartile Q3). The *p*-values were calculated using Welch t-tests or Wilcoxon rank-sum tests.

The primary endpoint was 30-day mortality. In the first step, a univariable Cox proportional hazards model was applied to evaluate the association between access site and the primary endpoint. Patients without events were censored at the earliest of re-surgery, the day of last observation, or the end of the observation period. Hazard ratios with 95% confidence interval (CI) and the *p*-value (H0: hazard ratio is equal to 1) were reported. Kaplan–Meier curves were used for graphical visualization of 30-day mortality, while a log-rank test was calculated to assess the difference in survival curves between the two groups.

For secondary time-to-event endpoints (e.g., Stroke), univariable cause-specific Cox regression models were fitted. Death was treated as a competing event, and individuals who died before the endpoint of interest were censored at the time of death. Patients who neither experienced the endpoint nor died were censored at the earliest of re-surgery, the day of last observation, or the end of the observation period. If the event occurred on the day of surgery, time-to-event was set to 0.5 days.

For major cardiac structural complications, a Cox regression model with Firth correction was applied due to complete separation [all events (*n* = 15) in the femoral group].

In the second step, multivariable Cox regression models were conducted, whereby access site, Euro Score II, and the variable systematic atherosclerotic vascular disease—which was defined as coronary arterial disease, peripheral vascular disease (PVD), and cerebrovascular disease (CVD)—were included as independent variables. Missing Euro Score II values were handled using multiple imputation based on predictive mean matching using the R-package mice (version 3.18.0, R-function mice, number of imputed datasets = 10). The following clinically relevant baseline characteristics were considered for multiple imputation: age, sex, insulin-dependent diabetes mellitus, chronic obstructive pulmonary disease, creatinine (mg/dL), NYHA class III–IV, CCS class, PVD, CVD, previous cardiac surgery, active endocarditis, LVEF >50%, and urgency. For each imputed dataset, Cox regression models were calculated, and estimates from each model were pooled into a single set of estimates and standard errors using Rubin's rules. If fewer than 15 events occurred, no multivariable model was applied.

The secondary endpoint of major vascular complications was analyzed as described earlier, but logistic regression models were applied instead of Cox regression models. It was assumed that major vascular complications were diagnosed within 2 days post-surgery in all patients. Further, intraprocedural outcomes were compared using Fisher's exact tests.

For overall survival, Kaplan–Meier estimates with 95% confidence limits for the time points 1, 3, and 5 years after surgery were provided. Length of hospital stay was analyzed descriptively, with the date of death being the discharge date for patients who died during hospital stay.

In addition, the proportion of patients with new conduction disturbances was compared between valve types (Navitor or Portico vs. remaining self-expandable valves) using Fisher's exact test in the total sample and in the two subgroups (TS, TF). Univariable Cox regression models were performed to estimate the association between postoperative pacemaker implantation and valve type in the total sample and in the two subgroups. For the subgroup TS, a Firth correction was applied due to complete separation.

A two-sided *p*-value of ≤ 0.05 was considered significant in all statistical tests. Due to the exploratory character of the study, no adjustment for multiplicity was done. Statistical analyses were performed using R statistical software, version 4.5.1.

## 3 Results

### 3.1 Baseline and procedural characteristics

All patients undergoing TAVR through transfemoral (*n* = 817) or transsubclavian (*n* = 75) access with a self-expanding prosthesis between August 2017 and May 2025 at our department were identified and included in the present analysis.

Baseline characteristics are presented in Table 1. Compared with the transfemoral group, patients who underwent TAVR through subclavian access were younger (79.53 ± 5.04 vs. 81.22 ± 6.45; *p* = 0.008), predominantly male (68.0% vs. 51.8%; *p* = 0.008), and had a higher median Euro Score II [5.00 (IQR: 2.48–8.23) vs. 3.13 (IQR: 1.90–5.50); *p* < 0.001]. The proportion of patients with atherosclerotic vascular disease was higher in the TS cohort, with PVD present in 57.3% (vs. 16.0%; *p* < 0.001), CVD in 42.7% (vs. 24.0%; *p* < 0.001), and coronary artery disease (CAD) in 56.0% (vs. 42.5%; *p* = 0.028). The TS cohort also demonstrated a markedly higher prevalence of previous myocardial infarction (25.3% vs. 11.5%; *p* = 0.002) and previous percutaneous coronary intervention (PCI) (44.0% vs. 30.0%; *p* = 0.018). Overall, atrial fibrillation/flutter was present in 35.9% of patients, while 13.9% had a history of cerebrovascular events (stroke/transient ischemic attack). The rate of end-stage chronic kidney disease was comparable between the two groups (6.7% vs. 2.6%; *p* = 0.06), while patients with transfemoral access had a significantly lower baseline creatinine [1.29 (IQR: 0.98–1.77) vs. 1.07 (IQR: 0.87–1.39); *p* < 0.001]. Preoperative echocardiographic findings showed reduced left ventricular function (EF < 50%) in 23.9% of the study cohort.

**Table 1:** Baseline characteristics.

Variables	Total *N* = 892 (100%)	TF-TAVR *N* = 817 (91.6%)	TS-TAVR *N* = 75 (8.4%)	*p*-value
Age (years), mean	81.07 ± 6.36	81.22 ± 6.45	79.53 ± 5.04	**0**.**008**
Sex: male	474 (53.1%)	423 (51.8%)	51 (68.0%)	**0**.**008**
BMI (kg/m^2^), mean number of missing values	26.99 ± 4.87 4	27.04 ± 4.84 3	26.49 ± 5.12 1	0.382
BSA (m^2^), mean number of missing values	1.86 ± 0.20 4	1.86 ± 0.20 3	1.88 ± 0.20 1	0.409
Euro Score II (%), median number of missing values	3.20 (1.94–5.66) 60	3.13 (1.90–5.50) 54	5.00 (2.48–8.23) 6	**<0.001**
STS score, median	8.93 (7.33–11.10)	8.93 (7.33–11.10)	8.82 (7.41–10.90)	0.916
NYHA class III–IV number of missing values	491 (60.8%) 85	446 (60.1%) 75	45 (69.2%) 10	0.185
Previous cardiac surgery	100 (11.2%)	90 (11.0%)	10 (13.3%)	0.565
Previous coronary revascularization	314 (35.1%)	278 (34.0%)	36 (48.0%)	**0.022**
CABG	70 (7.8%)	65 (8.0%)	5 (6.7%)	0.825
PCI	278 (31.2%)	245 (30.0%)	33 (44.0%)	**0.018**
Previous balloon valvuloplasty	5 (0.6%)	5 (0.6%)	0 (0.0%)	1.000
Porcelain aorta	20 (2.2%)	16 (2.0%)	4 (5.3%)	0.079
History of bleeding	82 (9.2%)	70 (8.6%)	12 (16.0%)	0.056
Diabetes mellitus	286 (31.9%)	256 (31.3%)	30 (40.0%)	0.154
Insulin-dependent	78 (8.7%)	67 (8.2%)	11 (14.7%)	0.083
Hyperlipidemia	691 (77.5%)	634 (77.6%)	57 (76.0%)	0.773
Hypertension	794 (89.0%)	728 (89.1%)	66 (88.0%)	0.702
COPD	152 (17.0%)	122 (14.9%)	30 (40.0%)	**<0.001**
Home oxygen use	17 (1.9%)	13 (1.6%)	4 (5.3%)	**0**.**047**
Coronary artery disease	389 (43.6%)	347 (42.5%)	42 (56.0%)	**0**.**028**
Previous myocardial infarction	113 (12.7%)	94 (11.5%)	19 (25.3%)	**0**.**002**
Peripheral vascular disease	174 (19.5%)	131 (16.0%)	43 (57.3%)	**<0.001**
Cerebrovascular disease	228 (25.6%)	196 (24.0%)	32 (42.7%)	**<0.001**
History of stroke or transient ischemic attacks	124 (13.9%)	112 (13.7%)	12 (16.0%)	0.600
Atrial fibrillation/flatter	320 (35.9%)	296 (36.2%)	24 (32.0%)	0.530
Congestive heart failure	251 (28.1%)	234 (28.6%)	17 (22.7%)	0.347
LVEF < 50% number of missing values	192 (21,5%) 90	171 (23.4%) 85	19 (27.1%) 5	0.46
Chronic kidney disease	285 (32.0%)	255 (31.2%)	30 (40.0%)	0.122
Dialysis	26 (2.9%)	21 (2.6%)	5 (6.7%)	0.060
Creatinine (mg/dL), median number of missing values	1.00 (0.88–1.40) 5	1.06 (0.87–1.39) 5	1.29 (0.98–1.71) 0	**<0.001**
Native bicuspid aortic valve	13 (1.5%)	10 (1.2%)	3 (4.0%)	0.088

Boldface indicates statistical significance (*p* ≤ 0.05).

For metric variables, mean ± SD and the *p*-value derived from the Welch test are reported if approximately normally distributed; otherwise, median (first quartile – third quartile) and the *p*-value derived from a Wilcoxon rank-sum test are shown. For categorical variables, absolute frequencies and percentages based on the number of valid observations and the *p*-value of the Fisher's exact test are shown.

BMI, body mass index; BSA, body surface area; STS, Society of Thoracic Surgeons; NYHA, New York Heart Association; CABG, coronary artery bypass grafting; PCI, percutaneous coronary intervention; COPD, chronic obstructive pulmonary disease; LVEF, left ventricular ejection fraction.

Procedural characteristics are presented in Table 2. Most of the procedures were performed in an elective setting. Preimplantation balloon dilatation was performed more frequently in the TS group (85.3% vs. 51.9%; *p* < 0.001), while no difference was observed in postimplant balloon dilatation (21.3% vs. 26.1%; *p* = 0.410). Navitor and Portico valves were used considerably more frequently in the TS group. Overall, 86.6% (65/75) of patients in the TS cohort received a Navitor or Portico valve, compared with 37.1% (303/817) in the transfemoral cohort.

**Table 2:** Procedural characteristics.

Variables	Total *N* = 892 (100%)	TF-TAVR *N* = 817 (91.6%)	TS-TAVR *N* = 75 (8.4%)	*p*-value
Urgency				0.37
Elective	858 (96.2%)	788 (96.5%)	70 (93.3%)	
Urgent	31 (3.5%)	26 (3.2%)	5 (6.7%)	
Emergent	3 (0.3%)	3 (0.4%)	0 (0.0%)	
Operative time (min), mean	61.94 ± 32.04	59.86 ± 31.59	84.55 ± 28.11	**<0.001**
Valve in Valve	63 (7.1%)	58 (7.1%)	5 (6.7%)	1.000
Combined procedures	11 (1.2%)	11 (1.3%)	0 (0.0%)	0.613
Valve type				**<0.001**
Portico	160 (17.9%)	138 (16.9%)	22 (29.3%)	
Navitor	208 (23.3%)	165 (20.2%)	43 (57.3%)	
Evolut R	130 (14.6%)	124 (15.2%)	6 (8.0%)	
Evolut Pro	61 (6.8%)	61 (7.5%)	0 (0.0%)	
Evolut Pro Plus	158 (17.7%)	154 (18.8%)	4 (5.3%)	
Evolut FX	156 (17.5%)	156 (19.1%)	0 (0.0%)	
Allegra THV	4 (0.4%)	4 (0.5%)	0 (0.0%)	
Symetis Acurate	4 (0.4%)	4 (0.5%)	0 (0.0%)	
Symetis Acurate Neo	11 (1.2%)	11 (1.3%)	0 (0.0%)	
Small valve size (≤23 mm)	64 (7.2%)	59 (7.2%)	5 (6.7%)	1.000
number of missing values	1	1	0	
Pre-dilatation	488 (54.7%)	424 (51.9%)	64 (85.3%)	**<0.001**
Post-dilatation	229 (25.7%)	213 (26.1%)	16 (21.3%)	0.410
Neuroprotection	639 (71.6%)	608 (74.4%)	31 (41.3%)	**<0.001**
Oral anticoagulation with NOAC (postoperative)	302 (34.2%)	280 (34.6%)	22 (30.1%)	0.520
Number of missing values	24	22	2	
Oral anticoagulation with VKA (postoperative)	24 (2.7%)	24 (3.0%)	0 (0.0%)	0.252
Number of missing values	24	22	2	
Single antiplatelet therapy (postoperative)	367 (41.6%)	340 (42.0%)	27 (37.0%)	0.458
Number of missing values	24	22	2	
Dual antiplatelet therapy (postoperative)	249 (28.2%)	224 (27.7%)	25 (34.2%)	0.225
Number of missing values	24	22	2	

Boldface indicates statistical significance (*p* ≤ 0.05).

Absolute frequencies and percentages are reported for categorical variables. The *p*-values have been calculated with Fisher's exact tests. Mean ± standard deviation was used to report metric variables. The *p*-value is derived from a Welch t-test. NOAC, non-vitamin K antagonist oral anticoagulants; VKA, vitamin K antagonist oral anticoagulants.

Compared with transfemoral TAVR, neuroprotection use was lower (41.3% vs. 74.4%, *p* < 0.001) and the procedural time was significantly longer (84.55 ± 28.11 vs. 59.86 ± 31.59, *p* < 0.001) with TS-TAVR. There were no significant differences in postoperative antiplatelet therapy and anticoagulation between the groups.

### 3.2 Primary outcome: 30-day mortality

**Figure 2:**
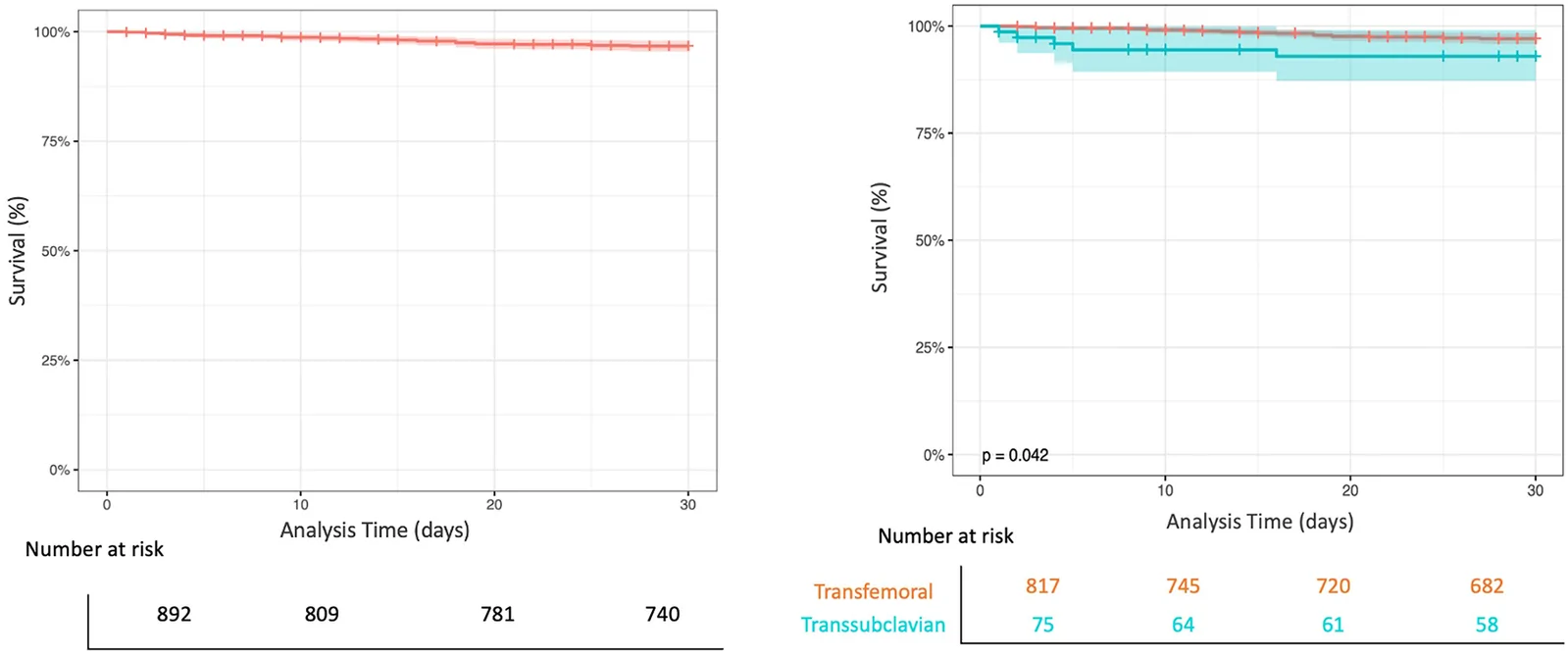
Kaplan–Meier analysis for all-cause mortality at 30 days. Left: total sample, Right: Blue: transsubclavian TAVR, Red: transfemoral TAVR. Survival at 30 days after TAVR is significantly higher in transfemoral TAVR compared with transsubclavian TAVR; log-rank *p* = 0.042.

Kaplan–Meier analysis (Figure 2) demonstrated a higher mortality rate at 30 days for patients undergoing subclavian access (6.7% vs. 2.7%; log rank *p* = 0.042). In total, we observed 27 deaths within 30 days after the index procedure. Univariable Cox regression analysis showed that the subclavian cohort was associated with a higher independent risk for 30-day mortality [HR 2.64, 95% CI: (1–6.97); *p* = 0.05]; however, after risk adjustment, the difference was no longer statistically significant [HR 2.27, 95% CI: (0.8–6.42); *p* = 0.12].

Survival at year 1-, 3-, and 5-year follow-up for patients undergoing TS-TAVR was 80.0% (69.7%; 91.8%), 76.4% (95% CI: 64.8–90.1%), and 67.9% (95% CI: 51.1–90.2%), while for TF-TAVR, it was 85.6% (82.8%; 88.5%), 63.4% (95% CI: 58.2–69.1%), and 39.7% (95% CI: 32.4%; 48.8%), respectively. These numbers should be interpreted cautiously, given the limited number of patients remaining at risk at later follow-up. Kaplan–Meier survival curves with numbers at risk are shown in the Supplementary Figure 1.

### 3.3 Secondary outcomes

In total, stroke was observed in 9 patients (12%) in the TS-TAVR cohort and in 45 patients (5.5%) in the TF-TAVR cohort over the entire observation period (censoring not considered). While most of the events were classified as ischemic stroke, only two patients undergoing transfemoral TAVR experienced hemorrhagic stroke. Myocardial infarction was observed in 3 patients (4%) in the TS-TAVR cohort vs. 9 patients (1.1%) in the TF-TAVR cohort. Cardiovascular hospitalization occurred in 19 patients (25.3%) in the TS group vs. 158 patients (19.3%) in the TF group. Major bleeding complications were observed in 25 patients (33.3%) in the TS-TAVR cohort and 167 patients (20.4%) in the TF-TAVR cohort. In the transfemoral cohort, 142 (17.4%) patients suffered from Type 2 bleeding, 20 patients (2.4%) from Type 3 bleeding, and 5 patients (0.6%) from Type 4 bleeding. Within the subclavian cohort, 22 (29.3%) patients experienced Type 2 bleeding and 3 patients (4.0%) experienced Type 3 bleeding. No Type 4 bleeding occurred with subclavian access. Overall, 111 patients (12.4%) received blood transfusions after TAVR, including 84 patients (10.3%) in the TF-TAVR group and 11 (14.6%) in the TS-TAVR group who required ≥2 units of red blood cells.

In univariable Cox regression analysis, TS-TAVR was associated with a significantly increased hazard for postoperative stroke [HR 2.44, 95% CI: (1.19–5.01), *p* = 0.01], myocardial infarction [HR 4.31, 95% CI: (1.16–15.96), *p* = 0.03], major bleeding [HR 1.79, 95% CI: (1.17–2.73), *p* = 0.007], and cardiovascular hospitalization [HR 1.65, 95% CI: (1.02–2.65), *p* = 0.04] compared with the TF-TAVR cohort. After adjustment for Euro Score II and systemic atherosclerotic disease in the multivariable Cox regression model, the observed associations with stroke and major bleeding remained statistically significant. Detailed results of the univariable and multivariable Cox regression analyses are presented in Table 3. For myocardial infarction, no multivariable Cox regression analysis was conducted given the limited number of events.

**Table 3:** Results of the univariable and multivariable Cox regression models adjusting for Euro Score II and systematic atherosclerotic vascular disease. For multivariable analysis, data imputation was used.

Endpoints	Univariable analysis		Multivariable analysis	
	HR (95% CI)	*p*-value	HR (95% CI)	*p*-value
30-day mortality	2.64 [1; 6.97]	0.05	2.27 [0.8; 6.42]	0.12
Stroke	2.44 [1.19; 5.01]	0.01	2.18 [1.03; 4.6]	0.042
Major bleeding	1.79 [1.17; 2.73]	0.007	1.69 [1.1; 2.61]	0.017
Cardiovascular hospitalization	1.65 [1.02; 2.65]	0.04	1.55 [0.96; 2.53]	0.076

Furthermore, we found no significant association between the access site and major vascular complications (9.3% vs. 7.6%, [OR 1.25, 95% CI: (0.55–2.85), *p* = 0.59]), major cardiac structural complications (0.0% vs. 1.8%; [HR 0.36; 95% CI: (0–2.7); *p* = 0.41]), acute kidney injury (11.6% vs. 12%; [HR 1.16; 95% CI: (0.58–2.3); *p* = 0.67]), and postoperative pacemaker implantation (17.5% vs. 12%; [HR 0.7, 95% CI: (0.36–1.38), *p* = 0.31]).

Overall, 69 patients (7.7% of overall cohort) experienced major vascular complications leading to VARC 3 Type ≥ 2 bleeding or limb ischemia. Aortic rupture during valve implantation occurred in 4 patients, vascular injury was documented in 39 (4.4%) patients, and closure device failure was observed in 38 (4.3%) patients. Only one patient in the subclavian cohort experienced suture failure with severe bleeding. As multiple mechanisms could coexist, these categories were not mutually exclusive. The spectrum of vascular injury included pseudoaneurysm formation (*n* = 13, 1.5%), stenosis or distal embolization leading to limb ischemia (*n* = 11, 1.2%), arterial or venous dissection or perforation (*n* = 12, 1.3%), hematoma or retroperitoneal bleeding (*n* = 8, 0.9%), and arteriovenous fistula (*n* = 3, 0.3%). Overall, 24 patients required endovascular or surgical intervention.

In the transfemoral group, major cardiac structural complications occurred in 15 patients, leading to VARC 3 Type ≥ 2 bleeding, hemodynamic compromise, tamponade, or the requirement for unplanned surgical or percutaneous intervention, including cardiac structural injury (*n* = 4), new pericardial effusion (*n* = 8), and coronary occlusion (*n* = 3). No major structural cardiac complications were observed in the transsubclavian cohort.

A total of 152 patients (17.0% of overall cohort) needed permanent pacemaker (PPM) implantation following TAVR. The incidence of new PM implantation did not differ significantly between groups. The use of Navitor or Portico valves was associated with a higher hazard of new permanent PM implantation, but this association was not statistically significant (total sample: Cox regression: HR 1.34 [95% CI: 0.97; 1.84], *p* = 0.07, TF: 1.39 [0.998; 1.93], *p* = 0.051, TS: Cox Regression with Firth Correction: 2.93 [0.37; 377.94], *p* = 0.38). In the TS group, only 9 patients received a PPM, while 65 out of 75 patients received a Navitor or Portico valve.

A detailed overview of clinical outcomes is provided in Supplementary Table 1.

### 3.4 Intraprocedural outcomes

Intraprocedural outcomes are presented in Table 4. No case of prosthesis malposition (0.0% vs. 2.9%; *p* = 0.253) was reported during valve implantation via the subclavian route. In total, 11 patients undergoing transfemoral approach required implantation of a second TAVR valve prosthesis during the index procedure. The rate of PVL (≥ moderate) was low in both groups (1.3% vs. 1.8%; *p* = 1). Admission to ICU was more frequent after TS-TAVR (16.0% vs. 8.4%; *p* = 0.036). LOS was 3 days (IQR: 2.0–7.5 days, maximum: 71 days) in the TS-TAVR cohort and 3 days (IQR: 2.0–6.0) maximum: 107 days) in the TF-TAVR cohort.

**Table 4:** Intraprocedural outcomes**.**

Variables	Total *N* = 892 (100%)	TF-TAVR *N* = 817 (91.6%)	TS-TAVR *N* = 75 (8.4%)	*p*-value
Valve malposition	24 (2.7%)	24 (2.9%)	0 (0.0%)	0.253
Implantation of > 1 valve	11 (1.2%)	11 (1.3%)	0 (0.0%)	0.613
Conduction disturbances	154 (17.3%)	150 (18.4%)	4 (5.4%)	**0**.**003**
PVL ≥ moderate	16 (1.8%)	15 (1.8%)	1 (1.3%)	1.000
Admission to ICU	81 (9.1%)	69 (8.4%)	12 (16.0%)	**0**.**036**

Boldface indicates statistical significance (*p* ≤ 0.05).

Absolute frequencies and percentages are reported, and the *p*-values have been calculated using Fisher's exact tests. PVL, paravalvular leak, ICU, intensive care unit, LOS, length of hospital stay.

Intraoperative conduction disturbances were observed more frequently in the TF cohort (18.4% vs. 5.4%; *p* = 0.003). Compared to other self-expandable valves, conduction disturbances occurred more often in patients undergoing TAVR with a Navitor or Portico valve (20.7% vs. 14.9%; *p* = 0.025). Subgroup analyses revealed that this difference was significant in the TF group, but not in the TS group (TF: 23.8% vs. 15.2%, Fisher's exact test: *p* = 0.003, TS: 6.2% vs. 0%, Fisher's exact test: *p* = 1).

## 4 Discussion

The question of optimal alternative access in patients unsuitable for transfemoral TAVR remains unresolved. Non-femoral arterial peripheral access gained popularity over years; however, despite its increasing use, available data regarding mortality and major clinical outcomes in TS-TAVR are inconclusive. While several studies have suggested favorable outcomes with subclavian access (8–10), a recently published large meta-analysis reported increased risk of all-cause mortality, stroke, myocardial infarction, and bleeding with TS-TAVR, highlighting the ongoing uncertainty regarding the optimal alternative access strategy in patients with hostile iliofemoral anatomy (11).

### 4.1 Early mortality

Although higher mortality rates in the TS-TAVR group were observed in our study, multivariable Cox regression analysis showed no significant association between access site and 30-day mortality. The increased mortality observed in the TS cohort likely reflects the elevated baseline risk profile of patients with subclavian access. An observational study by Yousef et al. revealed significantly higher mortality rates with TS-TAVR. Consistent with our findings, no significant association could be detected after multivariable risk adjustment (12).

Furthermore, two propensity score-matched analyses that compared transsubclavian and transfemoral approaches in TAVR with self-expandable valves demonstrated no significant association between subclavian access and death at 30 days (13, 14). Notably, van Wely et al. (14) included a single-center cohort of 354 patients undergoing TS-TAVR irrespective of femoral access suitability, thereby markedly reducing selection bias inherent to alternative access studies. These findings are further supported by two large-scale meta-analyses, showing comparable rates of early mortality between the transfemoral and transsubclavian approaches in patients undergoing TAVR (15, 16).

In contrast, the Spanish TAVR registry reported 2.3-fold increased odds of 30-day mortality in the subclavian cohort (17). In a retrospective data analysis by Grazina et al., transsubclavian TAVR and transcaval TAVR were associated with significantly lower survival rates at 30 days and 1 year compared with TF-TAVR (18).

### 4.2 Stroke

There is growing evidence that the risk for cerebrovascular adverse events is increased with the transsubclavian approach. A prior STS/ACC TVT Registry data analysis reported stroke rates as high as 14% at 1-year follow-up with the transsubclavian approach (19). Similarly, Grazina et al. showed a 30-day stroke rate of 13% in patients undergoing TS-TAVR. In the HOSTILE registry, patients with severe peripheral vascular disease who underwent non-thoracic transalternative access, predominantly subclavian, were associated with a higher risk of stroke/TIA at 30 days (5.9% vs. 2.8%) and 1 year (6.8% vs. 3.1%) compared with patients who underwent TF-TAVR with percutaneous transluminal angioplasty (20).

A large-scale meta-analysis by Faroux and colleagues demonstrated increased stroke rates with transsubclavian/transcarotid TAVR compared with the transfemoral approach. The increased risk of periprocedural stroke persisted after adjustment (21). Similarly, in the most recent meta-analysis by Yokoyama et al., TS-TAVR was significantly associated with higher stroke rates (11).

The underlying mechanisms for this observation remain unclear. A greater burden of vascular disease in patients requiring non-transfemoral access, together with the anatomical proximity of the subclavian artery to supra-aortic vessels, may be a possible explanation for higher stroke rates. The presence of cerebrovascular disease is an independent predictor of early stroke following TAVR (22). In our analysis, the subclavian cohort had a substantially higher prevalence of cerebrovascular disease. However, the association between access site and stroke remained statistically significant after adjusting for Euro Score II and systemic atherosclerotic vascular disease.

Furthermore, the presence of cerebrovascular disease may have influenced the use of neuroprotection devices in our study, as these patients might have been less anatomically suitable for cerebral embolic protection (CEP) use. In total, 74.4% of the patients undergoing transfemoral TAVR and 41.3% of the subclavian group received neuroprotection devices. Two recent RCTs report lower stroke rates with CEP use during TF-TAVR; however, the difference was statistically not significant (23, 24). Evidence regarding CEP use in alternative access TAVR is limited and warrants further investigation.

Procedural factors may have contributed to the higher stroke risk in the TS-TAVR group. There was significant heterogeneity in self-expandable valves used in both groups. Most patients undergoing TS-TAVR were treated with a Navitor or Portico valve (86.6%; *n* = 65), compared with only 37.1% (*n* = 303) in the TF-TAVR cohort. In the NORTHOSTAVI registry, balloon valvuloplasty—a known risk factor for periprocedural stroke following TAVR—was performed more frequently with the Navitor and Portico valve platforms (25). Similarly, we observed higher rates of pre-dilatation in the transsubclavian cohort, which may have increased the risk of cerebral embolization, thereby contributing to the higher stroke rate observed in our study.

### 4.3 Myocardial infarction and major cardiac complications

The mechanism underlying the higher incidence of myocardial infarction in TS-TAVR is not fully understood. Baseline comorbidity burden may be a possible explanation for the higher myocardial infarction rates in our study, as there was a higher prevalence of coronary artery disease and previous revascularization in the TS cohort. Some studies report higher periprocedural myocardial infarction rates due to intraprocedural cardiac structural complications disrupting coronary blood flow. According to van Wely et al., five out of eight patients with periprocedural myocardial infarction had a previous CABG with a patent LIMA graft at the time of intervention. LIMA graft overlying caused acute ischemia and hemodynamic instability in these patients (14). In contrast, three patients with patent LIMA grafts underwent subclavian TAVR at our department with no myocardial infarction at follow-up. Sheath size may also have influenced the outcomes. It must be noted that any acute or delayed coronary blood flow impairment related to valve or valve implantation in our study cohort was classified as cardiac structural complication in accordance with the VARC 3 criteria. Overall, three coronary obstructions (two acute, one delayed) occurred in the TF group. We observed no major cardiac structural complications in the subclavian cohort.

### 4.4 Major bleeding and major vascular complications

Concerns regarding higher risk of major bleeding and major vascular complications in patients undergoing TS-TAVR stem from the requirement for deep surgical dissection at the access site. The subclavian cohort in our study had a significantly higher risk of major bleeding, while major vascular complication rates were comparable between the groups. A previous report by Muscoli et al. showed overall bleeding rates of 35.3% and 9.4% in the TS- and TF-TAVR cohorts, respectively (26). In contrast, one of the largest meta-analyses by Ranka and colleagues reported no increased risk of major bleeding or major vascular complications within 30 days for TS-TAVR compared with TF-TAVR (10).

### 4.5 Conduction disturbances and new permanent pacemaker implantation

Several studies have suggested no increased risk of pacemaker implantation after TS-TAVR (14, 26). Bennes et al. (13) reported fewer new conduction disturbances with transsubclavian access, although this did not result in differences in postoperative pacemaker implantation. In contrast, Abusnina et al. (15) reported lower pacemaker implantation rates with the transfemoral approach.

We performed a subanalysis to evaluate whether valve type influenced the incidence of new conduction disturbances and permanent pacemaker implantation. Patients treated with a Navitor or Portico valve (*n* = 368) were compared with patients receiving other self-expandable valves (*n* = 524). Overall, patients undergoing TAVR with a Navitor or Portico valve had a higher risk of new intraprocedural conduction disturbances and permanent pacemaker implantations. The increased risk of conduction disturbances was statistically significant in the TF-TAVR group but not in the TS-TAVR group. A shorter implantation distance and a good coaxial alignment may have allowed a more controlled implant depth during TS-TAVR.

Consistent with our findings, Paukovitsch et al. demonstrated that, depending on valve size, the self-expanding Navitor valve was associated with significantly higher pacemaker implantation rates than the Evolut platform (adjusted OR 3.07, 95% CI 1.10–8.40) in patients undergoing TF-TAVR, while also showing that higher implantation depths substantially reduced the risk of permanent pacemaker implantation (27).

### 4.6 Acute kidney injury

Given the advanced age of patients undergoing TAVR, chronic kidney disease is common in this population, why contrast medium use during TAVR may further impair renal function and increase the risk of acute kidney injury (28). Two previously published studies reported lower incidences of acute kidney injury in the subclavian cohort. The authors suggested that this may be due to increased contrast medium use in transfemoral TAVR for angiographic control of the access site closure (29, 30). Our analysis demonstrated no association between access site and acute kidney injury.

### 4.7 Percutaneous TS-TAVR

All patients undergoing TS-TAVR at our department were treated via surgical cut-down. Percutaneous transsubclavian access, however, is increasingly adopted in contemporary practice. Current evidence suggests a faster postoperative recovery with percutaneous compared with surgical TS-TAVR, resulting in shorter ICU stays and reduced LOS, while maintaining comparable procedural success and early clinical outcomes (31, 32). A large propensity score-matched registry analysis by Chung et al. (32) reported a modest increase in major vascular complications. Increased vessel wall trauma during sheath exchange might cause higher rates of major vascular complications in percutaneous techniques compared with surgical cut-down.

Only a limited number of studies have directly compared percutaneous TS-TAVR and TF-TAVR. In a recently published single-center analysis, Corcione et al. reported comparable rates of all-cause mortality, stroke, myocardial infarction, major vascular complications, and major bleeding. Although limited by the small number of TS patients, these findings are encouraging and further support the feasibility of a fully percutaneous TS-TAVR (33).

## 5 Conclusion

The subclavian approach seems to be a feasible technique with an overall acceptable safety profile. In our study, multivariable Cox regression showed no statistically significant association with 30-day mortality. Patients in the subclavian cohort had a significantly higher operative risk and greater burden of atherosclerotic disease. Even after adjustment, TS-TAVR was significantly associated with stroke and major bleeding. However, residual confounding related to characteristics of patients not eligible for TF-TAVR cannot be fully excluded. Future research should focus on refining percutaneous techniques and evaluating the role of cerebral embolic protection and other neuroprotective strategies in TS-TAVR.

## 6 Limitations

The present study is inherently limited by its observational nature, where selection bias and baseline heterogeneity cannot be fully excluded. This is why our findings should be interpreted as hypothesis-generating rather than definitive evidence.

It should be acknowledged that the rates of blood transfusions were relatively high in both cohorts. This may reflect our institutional transfusion protocol, in which blood cell transfusion is generally initiated at hemoglobin levels of 8.0–8.5 g/dL. This approach should be considered when comparing our outcomes with those reported in other studies.

Furthermore, the subclavian cohort exhibited a substantially higher operative risk profile and greater prevalence of comorbidities compared with the femoral cohort. The higher stroke rates in the TS group may be attributable to the greater prevalence of cerebrovascular disease, more frequent balloon pre-dilatation, longer procedural times, higher utilization of Navitor and Portico valves, and the less frequent use of cerebral embolic protection devices.

Although we performed multivariable adjustment, only a limited number of covariates could be included in the model because of the relatively small number of events. Propensity score matching or other target trial emulation-based approaches like IPTW weighting were not performed, as group assignment was determined by anatomical characteristics assessed by CT scanning. Patients were only assigned to transsubclavian TAVR when femoral access was not feasible. Consequently, residual confounding cannot be excluded.

## Data Availability

Data supporting the findings of this study will be provided by the corresponding author upon request. Requests to access these datasets should be directed to Iuliana Coti, iuliana.coti@meduniwien.ac.at.
