# Supplementary material for: Transsubclavian versus transfemoral TAVR with self-expanding valves
Source: Front Cardiovasc Med. 2026 Aug 19;13:1901010. doi: 10.3389/fcvm.2026.1901010 (PMC13533891; doi:10.3389/fcvm.2026.1901010)
Supplement: Supplementary file 1 [file Supplementaryfile1.docx]

Supplementary Material

| **Supplementary Table 1. Outcome Variables in Detail** | | |
| --- | --- | --- |
| **Outcome** | **TF-TAVR**  **N = 817** | **TS-TAVR**  **N = 75** |
| 30-day mortality | 22 (2.7%) | 5 (6.7%) |
| Stroke | 45 (5.5%) | 9 (12%) |
| Myocardial infarction (MI) | 9 (1.1%) | 3 (4%) |
| Major bleeding  Type 2  Type 3  Type 4  Intrahospital blood transfusion  Red blood cell (RBC) ≥2 units | 167 (20.4%)  142 (17.4%)  20 (2.4%)  5 (0.6%)  99 (12.1%)  84 (10.3%) | 25 (33.3%)  22 (29.3%)  3 (4.0%)  0 (0.0%)  12 (16.0%)  11 (14.67%) |
| Major vascular complications  Aortic rupture  Vascular injury  Pseudoaneurysm  Dissection/Perforation  Arteriovenous fistula  Stenosis or distal embolization  Hematoma or retroperitoneal bleeding  Closure device failure  Endovascular/surgical intervention | 62 (7.6%)  4 (0.5%)  33 (4.0%)  13 (1.6%)  10 (1.2%)  2 (0.2%)  9 (1.1%)  6 (0.7%)  37 (4.5%)  20 (2.4%) | 7 (9.3%)  0 (0.0%)  6 (8.0%)  0 (0.0%)  2 (2.7%)  1 (1.3%)  2 (2.7%)  2 (2.7%)  1 (1.3%)  4 (5.3%) |
| Major cardiac structural complications (CSC)  Cardiac structural perforation  Pericardial effusion  Coronary obstruction | 15 (1.8%)  4 (0.5%)  8 (1.0%)  3 (0.4%) | 0 (0.0%)  0 (0.0%)  0 (0.0%)  0 (0.0%) |
| Pacemaker implantation (PM) | 143 (17.5%) | 9 (12%) |
| Acute kidney injury (AKI) | 95 (11.6%) | 9 (12%) |
| Cardiovascular hospitalization | 158 (19.3%) | 19 (25.3%) |

Figure 1: Kaplan Meier analysis for overall survival
